# Self-DNA acts as an immunostimulant for common bean (*Phaseolus vulgaris*) that induces defense against pests and diseases and increases seed yield

**DOI:** 10.1007/s44297-026-00086-3

**Published:** 2026-09-08

**Authors:** Dalia Durán-Flores, Martin Heil

**Affiliations:** Depto. de Ingeniería Genética, Centro de Investigación y de Estudios Avanzados (CINVESTAV) - Unidad Irapuato, Km 9.6 Libramiento Norte, 36421 Irapuato, GTO México

**Keywords:** Dry bean, Biological control, Damage-associated molecular patterns (DAMPs), Extracellular DNA, Induced defense, Yield improvement

## Abstract

Treating plants with plant-derived DNA triggers a strong and usually highly self/nonself-specific immune response. Several studies reported an induction of early signaling responses, resistance gene expression, and increased resistance to certain biotic stressors for self-DNA–treated Arabidopsis and also for crops like bean, lettuce, tomato, maize, or peach. These results indicate that self-DNA might bear potential as a tool for future strategies for a preventive biological pest control. However, it remains to be studied how DNA treatment affects different major defense hormones, to which degree responses at the early signaling levels predict the resistance at the phenotypic level, and whether the results of the earlier, lab-based studies would translate to the agronomic field. Here, we treated common bean (*Phaseolus vulgaris*) plantlets with self-DNA and with nonself-DNA from a congeneric species (lima bean, *Phaseolus lunatus*) and from a more distantly related species in the same family (*Acacia farnesiana*). We observed a self/nonself-specific induction of jasmonic acid only by self-DNA that was associated with decreased feeding by a chewing herbivore. By contrast, salicylic acid was induced by self- and nonself-DNA to similar degrees, which was also reflected in reduced population densities of several pathogenic fungi and bacteria. Most importantly, under open field conditions, a single self-DNA–treatment increased yield approximately 1.5-fold in the rainy season and 3.2-fold in the dry season. We suggest that self-DNA merits attention as a tool for future, preventive strategies in the biological control of pests and plant diseases.

**Supplementary Information:**

The online version contains supplementary material available at https://doi.org/10.1007/s44297-026-00086-3.

## Introduction

The world requires significant yield increases to feed an ever-increasing human population while also aiming to reduce pesticide use [1]. In this situation, “biostimulants” and “resistance elicitors” that activate plant immunity bear potential to replace curative, pesticide-based strategies of pest control with a preventive biological control. Here we aimed to investigate whether exogenously applied plant DNA stimulates the immune system of common bean (*Phaseolus vulgaris*), thereby increasing the resistance to pests and disease agents and, ultimately, yield. Common bean is a highly important staple crop, both in Mexico as its region of domestication and in many other regions of the world [2, 3]. The total production exceeds 23 million metric tons, with the largest producers being Brazil, Mexico, China, and the United States [4], but the true importance of common bean is that it contributes up to 15% of total daily calories and 36% of total daily protein to the diets of many subsistence-level farmers in Africa and Latin America (i.e., of more than half a billion people) [5, 6]. Nevertheless, common bean remains severely understudied with respect to novel alternatives to pesticide use.

For many years, plant growth–promoting rhizobacteria; fungi; extracts from seaweeds or plants; preparations of animal proteins; and laminarin, amino acids, humic acids, or chitosan have successfully been used as biostimulants of plant growth and nutrition. This effect is frequently associated with an induction of the plant’s immune system and, subsequently, increased resistance to biological threats, like disease agents and pests [7–13]. A molecule for which an immunogenic effect has been discovered more recently is self-DNA. Originally, DNA extracted from other plants of the same species was reported to cause the “Mazzoleni effect”: a dosage-dependent growth inhibition that seems to be a universal feature of plants and that is only observed after treatment with self-DNA, but not nonself-DNA from other plant species [14, 15]. Considering (a) that resistance induction usually comes at a cost that phenotypically becomes evident as a frequently transient growth reduction [16, 17], (b) the proposal that self-derived molecules can act as alarmins when they appear in fragments or inadequate compartments (i.e., Polly Matzinger’s danger model [18, 19]), and (c) the well-known immunogenic effects of DNA in mammals [20–23], we hypothesized that the Mazzoleni effect might result from a costly immune response to self-DNA [24].

Indeed, treatments with fragmented self-DNA activated early immune signals and the expression of defense-related genes in diverse plant species, including *Arabidopsis thaliana,* various algae, common bean and lima bean (*P. vulgaris* and *Phaseolus lunatus*), lettuce (*Lactuca sativa*), mesquite (*Prosopis laevigata*), tomato (*Solanum lycopersicum*), foxtail millet (*Setaria italica*), maize (*Zea mays*), rice (*Oryza sativa*), and even in harvested peach (*Prunus persica*) or loquat (*Eriobotrya japonica*) fruits [25–42]. Various studies reported increased phenotypic resistance to diverse biotic stressors, including thrips, aphids, necrotrophic and biotrophic fungi, oomycetes, and bacteria [28, 30, 42–45].

Studies that used self-DNA and nonself-DNA usually report stronger effects of self-DNA, even when using nonself-DNA from closely related species or different Arabidopsis ecotypes [28, 30, 31]. This level of self/nonself-specificity has not been reported for mammals. Moreover, overreactions of the mammalian immune system to its own DNA usually cause pro-inflammatory cell death, leading to severe inflammatory pathologies and autoimmune diseases [22, 46–49]. In the end, an immune response to self-DNA with positive net effects challenges the immunological paradigm of self-tolerance. Unfortunately, the lack of any known DNA receptor in plants represents a significant obstacle to understanding how these specific responses are generated, and important questions remain to be answered. Several studies reported DNA-induced gene expression patterns consistent with an activation of jasmonic acid (JA) signaling [32, 43, 50]. However, resistance to biotic stressors is controlled by two major hormones: JA controls the “wound response” that increases resistance to chewing herbivores and necrotrophic fungal pathogens, and salicylic acid (SA) controls systemic acquired resistance to sucking herbivores and biotrophic pathogens [51–54]. In most plants, these signaling pathways are subject to a negative trade-off [55, 56], which means that inducing the resistance to caterpillars might significantly increase the susceptibility to biotrophic bacteria and vice versa.

**Fig. 1:**
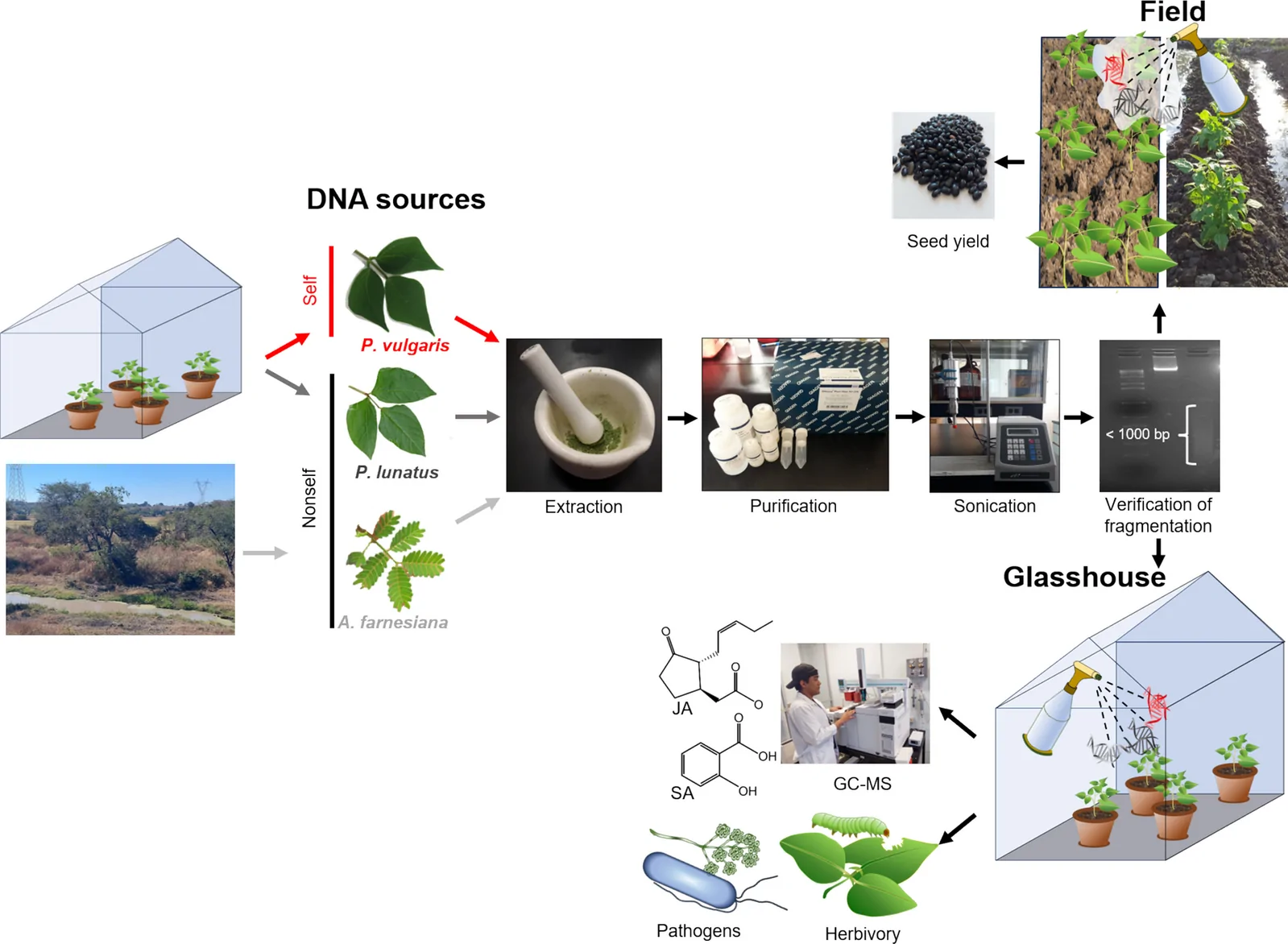
Methods summary of study workflow. Workflow shows which results have been obtained using plants in a glasshouse vs. field-grown plants. Abbreviations: *A.*, *Acacia*; GC–MS, gas chromatography—mass spectrometry; JA, jasmonic acid; *P.*, *Phaseolus*; SA, salicylic acid

Summarizing the aforementioned issues, self-DNA seems to be a promising candidate as a resistance inducer that might allow to grow plants without using pesticides. However, many details remain to be solved before its real-world application can be considered a realistic goal. Among others, it remained an open question whether SA is also induced by exogenously applied DNA, whether this induction is self/nonself-specific, and whether it is subject to the general SA-JA trade-off. In addition, it remained to be investigated as to which degree responses at the signaling or defense hormone level predict the phenotypic resistance to “real” relevant pests or pathogens. Finally, and most importantly from the agronomic perspective, we are not aware of a study that has quantified the net effects of DNA treatment on yield. Here, we combined glasshouse and field experiments in which *P. vulgaris* plants (cultivar Negro San Luis) were treated with self-DNA or with nonself-DNA from *P. lunatus* or *Acacia farnesiana*, aiming to close these knowledge gaps (see Fig. 1 for a graphical summary of the methods). We observed different responses of JA and SA, which were reflected in the phenotypic resistance to herbivores and pathogens, and which ultimately were associated with increased yield of self-DNA–treated plants, without any use of chemical pesticides. We suggest that self-DNA merits attention as a tool for future, preventive strategies in the biological control of pests and plant diseases.

## Materials and methods

### Plants and DNA sources

We used four-week-old common bean (*P. vulgaris* L.) plants (variety Negro San Luis) grown in pots in a glasshouse for the quantification of defense hormones and biological resistance and as sources of self-DNA; *P. vulgaris* plants grown under field conditions were used for the quantification of seed production as an approximation of fitness (Fig. 1). Seeds of this bean variety had been obtained from the national germplasm collection at the National Institute for Forestry, Agriculture and Livestock Research (INIFAP) in Celaya, Guanajuato, Mexico, and were used to establish a population in a glasshouse at Centro de Investigación y de Estudios Avanzados (CINVESTAV) Irapuato that is continuously being used for propagation. All seeds for the present study were collected from this population. Before sowing, the seeds were surface-sterilized with 70% ethanol for 1 min and 20% hypochlorite for 10 min, and then they were washed five times with sterile distilled water. Conditions in the glasshouse were natural light, natural photoperiod, an average daytime temperature of 28 °C, and an average nighttime temperature of 20 °C. Plants were cultivated in pots with a volume of 10 L, filled with a custom-made substrate composed of 1.8 kg of peat moss, 1.2 kg leaf soil, 0.6 kg dolomitic limestone, 0.6 kg de perlite (Termolita, Nuevo León, Mexico) and 0.6 kg vermiculite (Sun Gro Horticulture, Bellevue, Washington, USA); they were watered three times a week and fertilized weekly with a commercial fertilizer (Ferviafol 20–30-10, Agroquímicos Rivas, Celaya, Guanajuato, Mexico). The experimental field was located at CINVESTAV Irapuato, Guanajuato, Mexico (approx. 20° 43′ 13″ N and 101° 19′ 43″ W, 1730 m above sea level).

As sources of nonself-DNA, we used leaves of lima bean (*P. lunatus* L.) plants grown in the same glasshouse from seed collected from a wild population 5 km west of Puerto Escondido, Oaxaca, Mexico (approx. 15° 55′ N and 97° 09′ W) and leaves of wild *A. farnesiana* (L.) Willd. plants growing in the area around CINVESTAV Irapuato.

### Herbivore

Larvae of the generalist herbivore *Spodoptera frugiperda* (J.E. Smith) were collected from maize fields in Irapuato, Guanajuato, Mexico, identified by following an established taxonomic key [57], and reared in individual 100 mL plastic cups with perforated lids, allowing ventilation. They were on an artificial diet (1–2 cm^3^) prepared with 12.8% of corn semolina, 3.2% of wheat germ, 3.4% of brewer yeast, 0.45% of ascorbic acid, and 0.13% of benzoic acid dissolved in 1.8% agar aqueous solution and allowed to solidify. The adult insects were provided with a 10% v/v^−1^ of honey solution in a wet cotton ball. The colony was maintained until the third generation to increase the probability that the larvae were parasite free. For the herbivory experiment, we used larvae at the fifth instar (14 days after oviposition) that had been maintained for 12 h without food before starting the experiment.

### Phytopathogens

The bacterium *Xanthomonas axonopodis* pv. *phaseoli* was provided by Dr Gabriel Gallegos-Morales (Universidad Autónoma Agraria Antonio Narro Oficial in Saltillo, Coahuila, Mexico) and cultivated at 28 °C on solid yeast dextrose carbonate medium [58]. *Pseudomonas syringae* pv. *phaseoli* strain NPS3121 was provided by Dr José-Luis Hernández-Flores (CINVESTAV Unidad Irapuato in Irapuato, Guanajuato, Mexico) and cultivated at 28 °C on solid King’s B medium [59]. The rifampicin-resistant *P. syringae* pv. *syringae* strain 61 was provided by Dr Choong-Min Ryu (Korea Research Institute of Bioscience and BioTechnology in Daejeon, South Korea) and cultivated at 28 °C on solid King’s B medium with 50 mg L^−1^ rifampicin. The *Enterobacter* sp. strain FCB1 had previously been isolated and identified in our laboratory [60] and was cultivated at 28 °C on solid yeast dextrose carbonate medium. The fungal pathogens we use were as follows: *Colletotrichum lindemuthianum* strain 1088 was donated by Dr June Simpson (CINVESTAV Unidad Irapuato), *Sclerotinia sclerotiorum* was donated by Dr Victor Olalde-Portugal (CINVESTAV Unidad Irapuato), and *Fusarium oxysporum* and *Botrytis cinerea* were provided by Dr Alfredo Herrera-Estrella (CINVESTAV Unidad de Genómica Avanzada in Irapuato, Guanajuato, Mexico). All fungi were cultivated on plates containing potato dextrose agar medium at 28 °C except for *S. sclerotiorum*, which was cultivated at 20 °C.

### Extraction and fragmentation of DNA

The extraction of DNA from leaves of *P. vulgaris*, *P. lunatus*, and *A. farnesiana* was based on a method reported by Dellaporta et al. [61]. Leaves of *P. vulgaris*, *P. lunatus*, or *A. farnesiana* were ground with liquid nitrogen. A total of 20 mL of Dellaporta buffer (100 mM Tris–HCl pH 8, 5 mM EDTA pH 8, 50 mM NaCl, and 10 mM β-mercaptoethanol) were added per 5 g of ground tissue in 50 mL tubes, shaken on a vortex and heated to 65 °C for 10 min in a water bath. Then, 6.6 mL of 5 M potassium acetate were added followed by incubation on ice for 30 min. The tubes were centrifuged at 12,000 g for 20 min at 4 °C: the supernatant was transferred to a new 50 mL tube. Next, 20 mL of precooled isopropanol was added to the supernatant, which was kept at − 20 °C. After 1 h, the tubes were centrifuged at 12,000 × *g* for 20 min at 4 °C, the supernatant was discarded, and the pellet was dried for 5 min at room temperature before washing by adding 5 mL of 70% ethanol and shaking. After further centrifugation at 12,000 × *g* for 10 min a 4 °C, the supernatant was discarded and the pellet was dried for 5 min and suspended in 1 mL of sterile distilled water and purified using a Maxi DNA purification Kit (Qiagen, Hilden, North Rhine-Westphalia, Germany).

The DNA was quantified using a NanoDrop 2000 spectrometer (Thermo Fisher Scientific, Waltham, Massachusetts, USA) and fragmented by sonication using an ultrasonic processor (Misonix XL2020, Misonix Incorporated, Farmingdale, New York, USA). To obtain fragments shorter than 1000 bp, a solution of 500 μg mL^−1^ of DNA in sterile distilled water was sonicated for 3 min at 55% of amplitude and using pulse mode (1 s pulse “On” and a 1 s pulse “Off”). The successful fragmentation of DNA was verified by gel electrophoresis on a 3% agarose gel using ethidium bromide staining.

### JA and SA levels

To quantify the effects of exogenously applied self-DNA on the synthesis of JA and SA, we applied fragmented self-DNA at different concentrations (0, 2, 20, 50, 100, 150, or 200 µg mL^−1^ in 0.05% v/v^−1^ Tween 20). Using a micropipette, the solution of self-DNA was applied to both sides of the three youngest leaves of each plant until the leaf surface was completely wet (approx. 8–10 mL per plant). Independent groups of six plants were used for each time point and for each self-DNA concentration. Treated leaves were cut off at the base at 0, 2, 5, 10, 15, 30, or 45 min or at 1, 2, 4, 8, 12, 24, or 48 h after treatment; the three leaves from each plant were pooled and ground with liquid nitrogen. To compare the effects of self and nonself-DNA, we used 50 µg mL^−1^ of DNA to treat groups of six plants and quantified JA 30 min after treatment, whereas SA was quantified 24 h after treatment.

The extraction of JA followed Pluskota et al. [62] and the extraction of SA followed Malamy et al. [63] and Meuwly and Metraux [64]. In brief, to extract JA, 0.5 mL of ethyl acetate and 20 µl of 0.1 mg mL^−1^ (±)-dihydrojasmonic acid (Merck no. CDS022683, purchased from Sigma-Aldrich, St. Louis, Missouri, USA) as internal standard were added to 250 mg of tissue, shaken, and kept at 4 °C overnight. After centrifugation at 14,000 × *g* for 15 min at 4 °C, the supernatant was collected, and the pellet was re-extracted with 0.5 mL of ethyl acetate and centrifuged. The supernatants were combined and completely evaporated with gaseous nitrogen. The residue was derivatized [65] by adding 100 µL of N,N-diisopropylethylamine, 100 µL of chloroform, and 10 µL of pentafluorobenzyl bromide (all from Sigma-Aldrich), and keeping the mixture at 60 °C. After 30 min, the resultant liquid was cooled on ice and completely evaporated with gaseous nitrogen. The residue was resuspended with 100 µL of HPLC grade methanol.

To extract SA, we added 750 µl of 90% methanol and 250 ng mL^−1^ of ortho-anisic acid (internal standard) to 250 mg of ground tissue and kept at 4 °C overnight. After centrifugation at 13,000 × *g* for 15 min at 4 °C, the supernatant was collected, the pellet was re-extracted with 750 μl of 90% methanol, and both supernatants were combined and dried in a Centrifuge Concentrator Plus (Eppendorf, Hamburg, Germany) for 4 h. The residue was resuspended with 500 μl of 5% trichloroacetic acid and centrifuged at 4000 × *g* for 10 min at 4 °C. The supernatant was mixed with two volumes of ethyl acetate-hexane (1:1 v/v^−1^) and incubated at room temperature for 10 min. The organic phase (upper phase) was recovered and dried with gaseous nitrogen. The residue was derivatized by mixing with 20 µl of pyridine and 80 μl of N,O-bis(trimethylsilyl)trifluoroacetamide (Sigma-Aldrich) and incubated at 80 °C for 1 h using an extraction hood.

Both hormones were quantified using gas chromatography coupled to an electronic impact ionization mass spectrometer in the Gas Chromatograph 7890 A equipped with a DB-1 MS Ultra Inert Column (60 m × 0.25 mm × 0.25 µm, Agilent Technologies, Santa Clara, CA, USA) coupled to a mass selective detector (MSD 5973, Agilent Technologies) in selective ion modus using ions 141, 181, 390, and 392 m z^−1^ for JA and 73, 135, 267, and 282 m z^−1^ for SA. An injection volume of 1 µl of sample was used in the splitless mode. For JA, the operating conditions followed Ramírez-Chavez et al. [66]: we used an injector temperature of 200 °C and an initial oven temperature of 150 °C for 3 min, which was then ramped at 4 °C min^−1^ to 300 °C, with the final temperature maintained for 20 min. For SA, we used an injector temperature of 200 °C and an initial oven temperature of 150 °C for 3 min, which was then ramped at 4 °C min^−1^ to 260 °C, with the final temperature maintained for 25 min. Helium was used as a carrier gas with a constant flow of 1 mL min^−1^, and standard curves were prepared using pure compounds (JA, Sigma-Aldrich; SA, J. T. Baker, Phillipsburg, New Jersey, USA) to quantify the respective amounts of JA and SA based on peak areas with reference to the internal standard.

### Herbivore feeding

Plants of *P. vulgaris* were treated with 50 µg mL^−1^ of self- or nonself-DNA as described above (*n* = 7 for each DNA source). After 30 min, one larva of *S. frugiperda* was placed on each leaf, which was then covered with a mesh bag. After 24 h, individual photos of each leaf including a ruler at one side were taken using a J5-Prime Samsung phone camera from 20 cm distance. Feeding damage was quantified as percentage of lost area using ImageJ software (https://imagej.net/ij/) to measure the lost area by herbivory.

### Bacterial and fungal infection

Plants of *P. vulgaris* were treated with 50 µg mL^−1^ of self- or nonself-DNA as described above (*n* = 10 for each DNA source and pathogen). Five minutes after the treatment, plants were sprayed with a 10 mL suspension (1 × 10^7^ cells mL^−1^) of one of the seven pathogens. Infection levels of three randomly selected leaves per plant were quantified after 8 days (bacteria) or 15 days (fungi). Leaves were collected, weighed, and ground in a mortar with 1 mL of sterile distilled water, diluted 1:100 in sterile distilled water, and 20 µl of the resultant liquid were plated onto the appropriate solid medium as described in the “Phytopathogens” section to quantify colony-forming units (CFUs). The putative direct effects of DNA solutions on pathogens [67] were investigated by plating 100 µl of 50 µg mL^−1^ of DNA of either *P. vulgaris*, *P. lunatus*, or *A. farnesiana* DNA (*n* = 6 for each DNA type and pathogen) on Petri dishes of solid medium. After 5 min, the medium was inoculated with the respective pathogen and CFUs were quantified 2 days after inoculation for bacteria and 4 days after inoculation for fungi.

### Seed production

Plants of *P. vulgaris* were cultivated in experimental fields at CINVESTAV Irapuato in 12 blocks (3 per treatment) of 3 × 9 plants, with 35 cm between individuals and 1 m between blocks (see Fig. S2). This experiment was carried out from April to July of 2018 during the early rainy season, which is the main cultivation period for *P. vulgaris* in this region [68]. The experiment was repeated from July to October of 2018. Four-week-old plants were treated by spraying 8 to 10 mL of a solution of 50 µg mL^−1^ of self- or nonself-DNA in 0.05% v/v^−1^ Tween 20 on both sides of all leaves until the plants were completely wet. Plants treated with 0.05% Tween 20 were used as controls [69]. Plants were allowed to finish their growing cycle with no further treatment. Pods were collected from the seven central plants of each block and the total mass of seeds from each plant was recorded.

## Results

### Self-DNA induces JA and SA

**Fig. 2:**
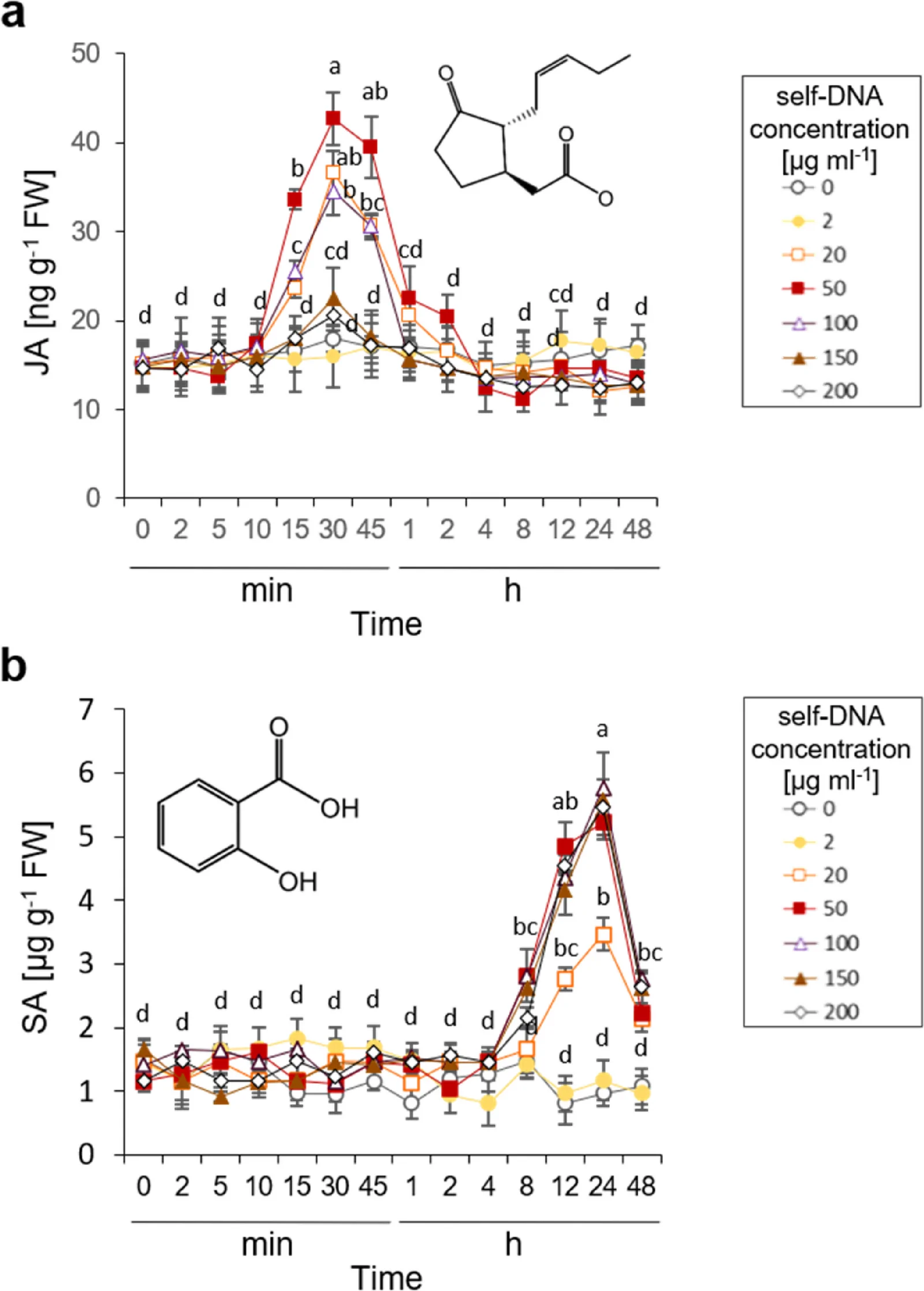
Induction of plant defense hormones by self-DNA. Common bean plants were treated with different concentrations of self-DNA and samples were taken at different time points to quantify the concentration of (**a**) JA in nanograms of JA per grams of leaf FW and of (**b**) SA in micrograms per gram of leaf FW. Symbols on colored lines indicate means ± SE; each symbol indicates the different concentrations of self-DNA used, and symbols marked with different letters indicate significant differences among the respective treatments at each specific time point (univariate ANOVA and post hoc Tukey test; *P* < 0.05, *n* = 6). Abbreviations: ANOVA, analysis of variance; FW, fresh weight; JA, jasmonic acid; SA, salicylic acid; SE, standard error

We aimed to elucidate whether the specificity observed in the early, local immune signaling responses to DNA also affects systemic, hormone-based defense signaling. Therefore, we first characterized the effects of a treatment with exogenously applied self-DNA (extracted from other *P. vulgaris* plants of the same cultivar [28]) on the plant hormones, JA and SA. Self-DNA induced both hormones in *P. vulgaris* plantlets, although JA and SA increased at different times after treatment and showed different dose–response relationships (Fig. 2). Endogenous JA levels started to increase at 15 min and peaked at 30 min after the application of self-DNA, reaching maximum levels that depended significantly on the used self-DNA concentration (Fig. 2a, general linear model [GLM]; *P* < 0.001, *n* = 6). This dose–response relation followed an optimum curve: although concentrations of 20 μg mL^−1^, 50 μg mL^−1^, or 100 μg mL^−1^ of self-DNA had significant effects (Fig. 2a, GLM; *P* < 0.001, Tukey post hoc test; *P* < 0.05, *n* = 6), we could detect no significant effects of lower (2 μg mL^−1^) and higher concentrations (150 μg mL^−1^ and 200 μg mL^−1^; GLM, Tukey post hoc test;* P* < 0.05, *n* = 6). In short, we observed the strongest JA induction (to levels > 40 ng JA g^−1^ fresh weight) at 30 min after treatment with 50 μg mL^−1^ self-DNA (Fig. 2a).

Self-DNA also induced SA synthesis in a strongly time- and concentration-dependent manner (Fig. 2b, GLM; *P* < 0.05, *n* = 6). SA levels started to increase at 8 h and peaked at 24 h after the application of self-DNA, and the dose–response relation followed a saturation curve: the minimum concentration of self-DNA that induced a statistically significant increase in endogenous SA levels was 20 μg mL^−1^ (GLM, Tukey post hoc test; *P* < 0.001), and all higher concentrations tested (50 μg mL^−1^, 100 μg mL^−1^, 150 μg mL^−1^, and 200 μg mL^−1^) induced maximum levels of approximately 5 μg SA g^−1^ fresh weight (Fig. 2b, GLM; *P* < 0.001, Tukey post hoc test; *P* < 0.05, *n* = 6). Based on these results, we selected 50 μg mL^−1^ DNA as the optimal concentration for all subsequent experiments.

### Self/nonself-specific effects of DNA on JA and SA

**Fig. 3:**
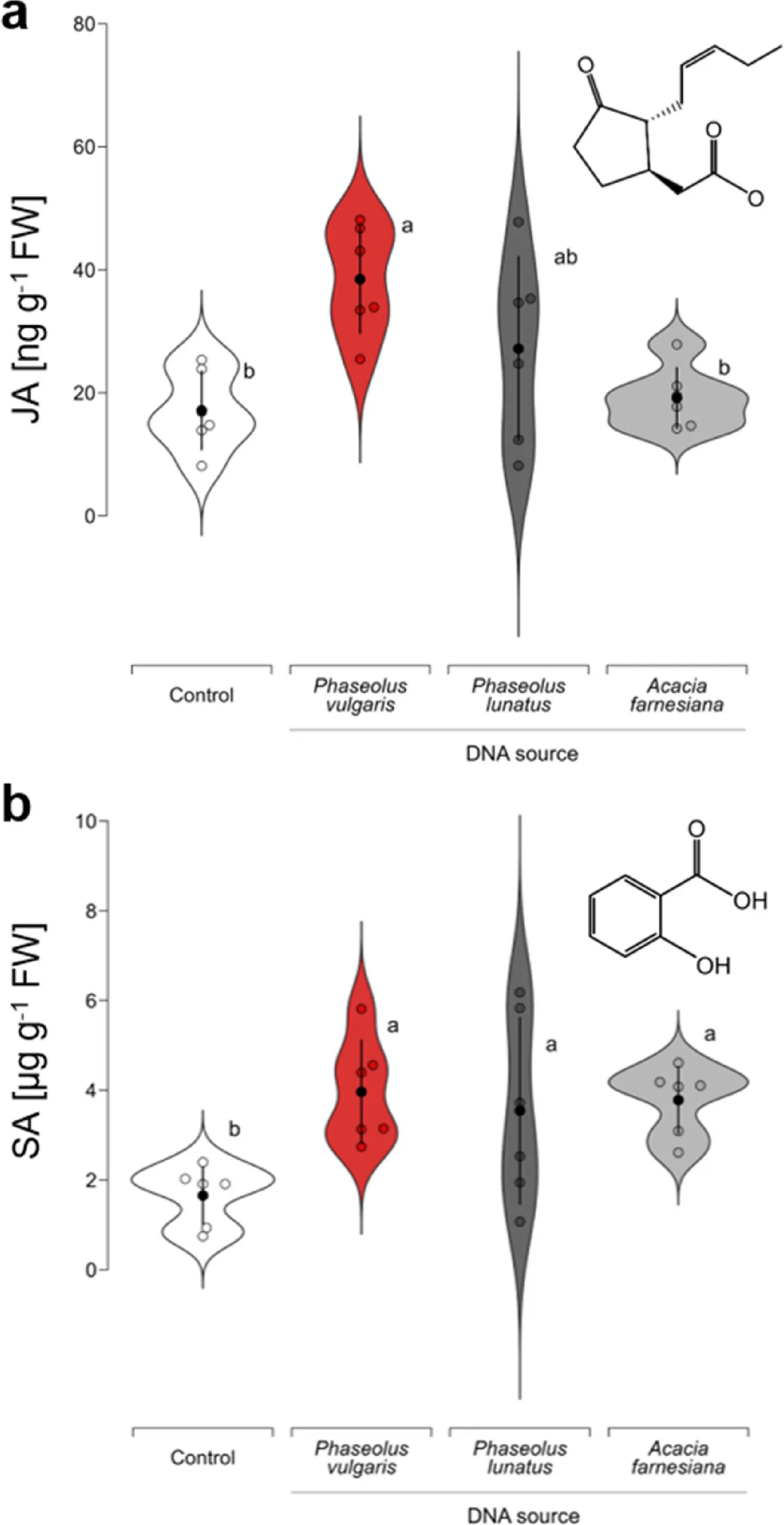
Self/nonself-specific induction of JA and vs. nonspecific induction of SA. Violin plots in **a** show the frequency distribution of the concentration of JA in nanograms of JA per gram of leaf FW at 30 min after treatment, and **b** shows the concentration of SA in micrograms per gram of leaf FW at 24 h after treating common bean plants with 50 µg mL^−1^ of self-DNA (from *Phaseolus vulgaris* [red]) or nonself-DNA (from *Phaseolus lunatus* [dark gray] and *Acacia farnesiana* [light gray]) or 0 μg mL^−1^ of DNA (control [white]). Insets within violins indicate mean (black dots) ± 1 SD; different letters indicate significant differences among treatments (univariate ANOVA and post hoc Tukey test; *P* < 0.05, *n* = 6). Abbreviations: ANOVA, analysis of variance; FW, fresh weight; JA, jasmonic acid; SA, salicylic acid; SD, standard deviation

Next, we tested for self/nonself-specific differences in the response of both hormones to exogenous DNA. Aiming to detect a potential phylogenetic signal [30, 31], we prepared nonself-DNA from *P. lunatus* and *A. farnesiana*, two plant species belonging to the same family as *P. vulgaris* that had been used earlier as sources of nonself-DNA [28]. When we treated *P. vulgaris* plantlets with self or nonself-DNA at 50 μg mL^−1^ (or with water with 0.05% v/v^−1^ Tween 20 as a control) and quantified endogenous JA at 30 min after treatment, we observed a statistically significant increase in JA only in response to self-DNA (Fig. 3a, GLM, Tukey post hoc test; *P* < 0.01, *n* = 6). Nonself-DNA from *P. lunatus* also seemed to increase JA, although this could not be confirmed statistically (GLM, Tukey post hoc test; *P* > 0.05, *n* = 6), whereas nonself-DNA from *A. farnesiana* had no detectable effect at all (GLM, Tukey post hoc test; *P* > 0.05, *n* = 6). In contrast to this self/nonself-specific JA response, all three types of DNA caused a significant induction of SA (GLM; *P* < 0.001, *n* = 6), with no statistically significant differences between the types of DNA (Fig. 3b, GLM, Tukey post hoc test; *P* > 0.05, *n* = 6, for each DNA type vs control).

### Exogenous DNA triggers self/nonself-specific resistance to a chewing herbivore

**Fig. 4:**
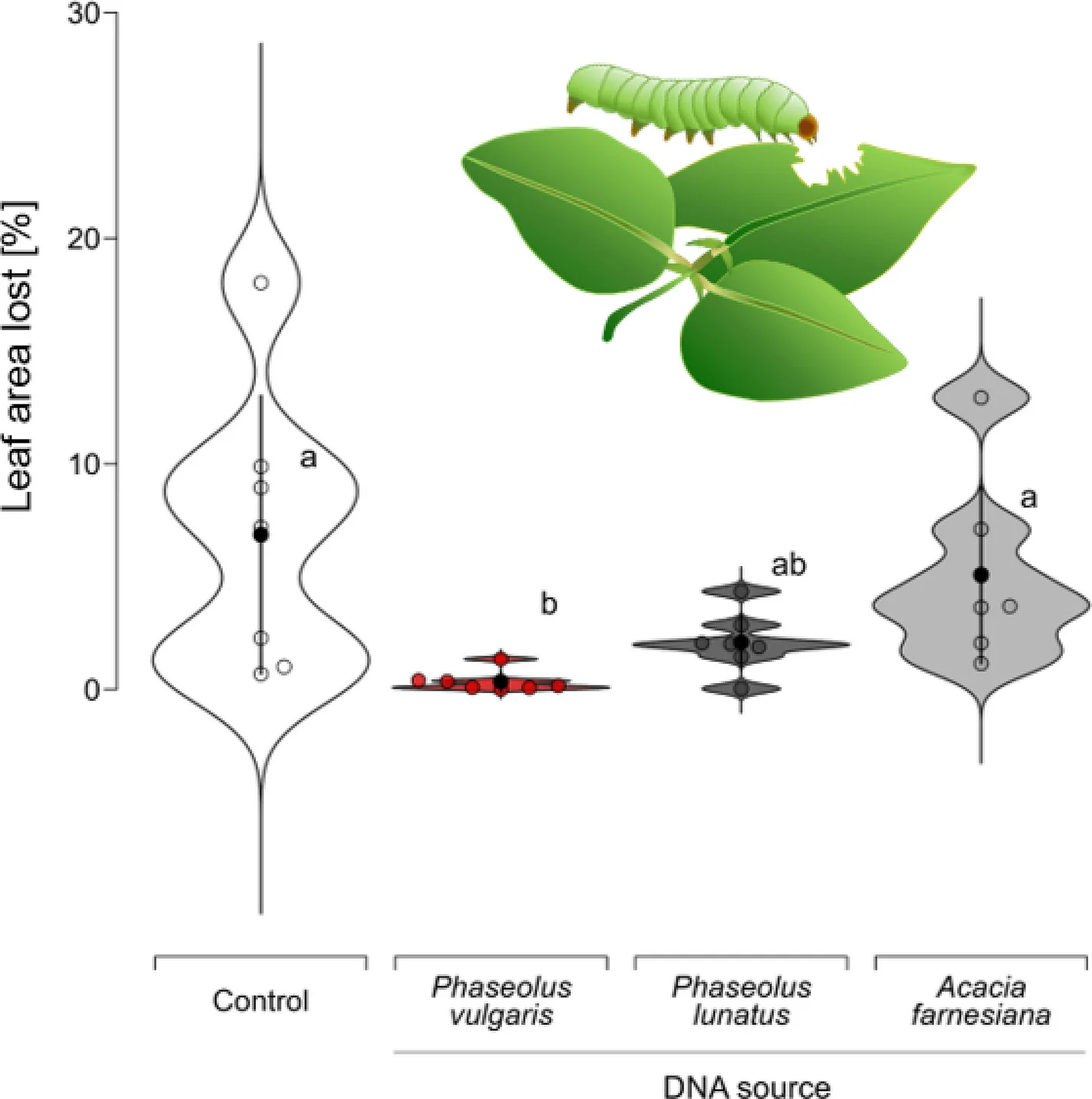
Self/nonself-specific induction of resistance against a chewing herbivore. Violin plots depict the frequency distribution of leaf area removed by *Spodoptera frugiperda* larvae feeding during 24 h on one leaf. There was one *Phaseolus vulgaris* leaf per larva, after treatment with 50 µg mL^−1^ of self-DNA (*Phaseolus vulgaris* [red]) or nonself-DNA (*Phaseolus lunatus* [dark gray] and *Acacia farnesiana* [light gray]). The white violin represents the control (0 µg mL^−1^ of DNA). Insets within violins indicate mean (black dots) ± 1 SD; different letters above bars indicate significant differences among treatments (univariate ANOVA and post hoc Tukey test; *P* < 0.05, n = 7). Abbreviations: ANOVA, analysis of variance; SD, standard deviation

In most plants, JA controls the defense against chewing herbivores and necrotrophic pathogens, whereas SA controls resistance to sucking herbivores and biotrophic pathogens; usually, both signaling pathways are subject to a negative trade-off [53, 70]. Nevertheless, exceptions to this general model are common [55]. Therefore, we decided to complement the quantification of hormones with bioassays using a diverse spectrum of plant enemies. We selected *S. frugiperda* as a chewing herbivore, treated bean plantlets with 50 µg mL^−1^ of self- or nonself-DNA, and quantified the percentage of leaf area consumed by one caterpillar that was feeding on one leaf over the following 24 h. We observed a significant treatment effect (Fig. 4, GLM; *P* < 0.05, *n* = 7). However, of the three DNA types tested, only self-DNA reduced leaf area loss significantly, from approximately 7% in controls to approximately 0.3% in treated plants (Tukey post hoc test; *P* < 0.05, *n* = 7). Nonself-DNA from *P. lunatus* reduced leaf area loss to approximately 2%, although this effect was not statistically significant (GLM, Tukey post hoc test; *P* > 0.05, *n* = 7), and nonself-DNA from *A. farnesiana* had no detectable effect (Fig. 4, GLM, Tukey post hoc test; *P* > 0.05, *n* = 7).

### Exogenous self- and nonself-DNA reduce infection by fungal and bacterial pathogens

**Fig. 5:**
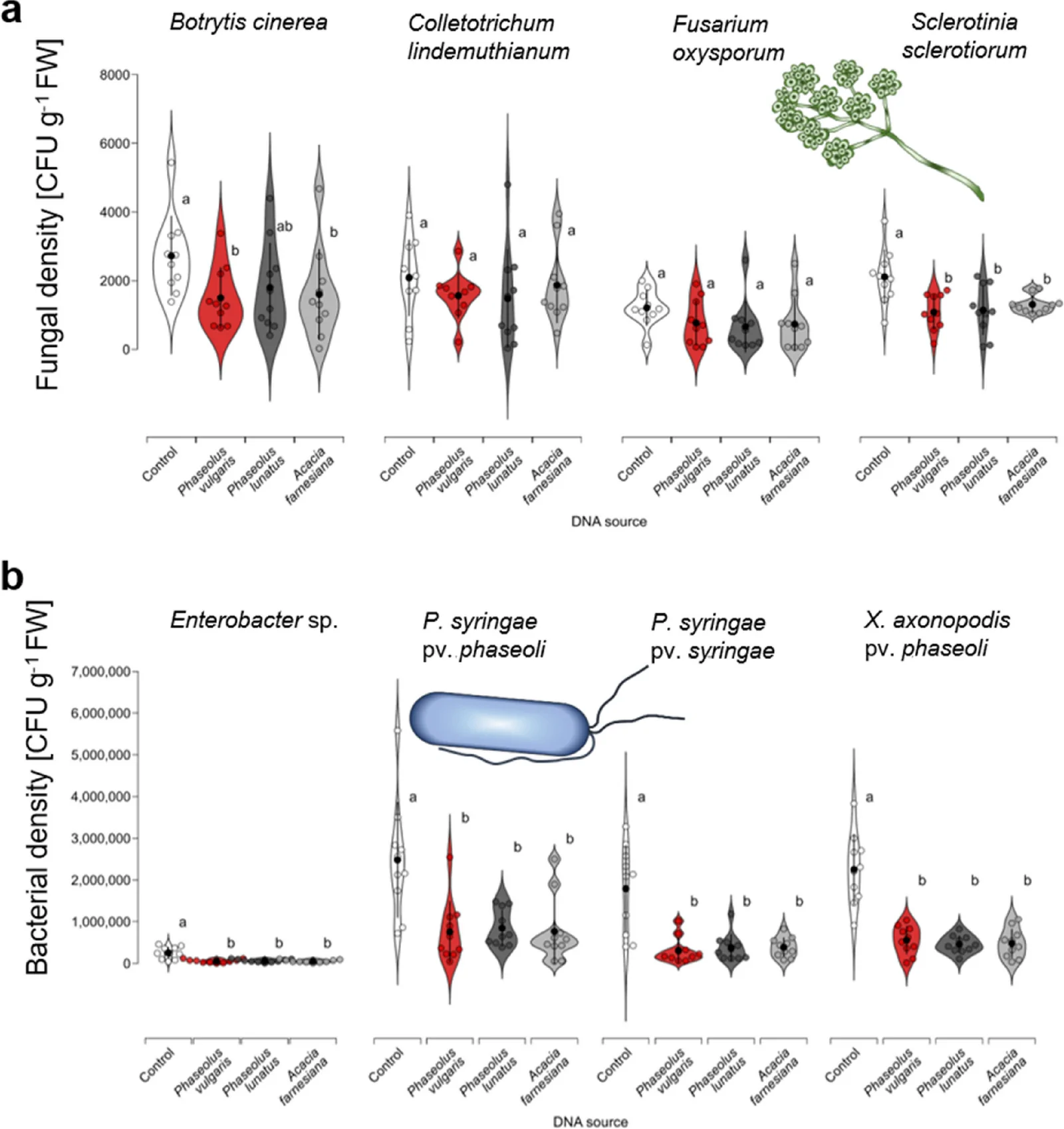
Self- and nonself-DNA treatment reduces infection by bacterial and fungal pathogens. Violin plots depict the frequency distribution of CFUs of (**a**) the fungal pathogens *Botrytis cinerea, Colletotrichum lindemuthianum*, *Fusarium oxysporum*, and *Sclerotinia sclerotiorum* and of (**b**) the bacterial pathogens *Enterobacter* sp. strain FCB1, *Pseudomonas syringae* pv. *phaseoli*, *P. syringae* pv. *syringae*, and *Xanthomonas axonopodis* pv. *phaseoli* in *Phaseolus vulgaris* plants that had been treated with 50 µg mL^−1^ of self-DNA (*Phaseolus vulgaris* [red]) or nonself-DNA (*Phaseolus lunatus* [dark gray] and *Acacia farnesiana* [light gray]). Samples for CFU determination were taken at 8 days (bacteria) and 15 days (fungi) after inoculating the plants, and CFUs were counted after 2 days (bacteria) or 4 days (fungi) after plating leaf homogenates onto the appropriate solid medium. The white violins represent the controls (0 µg mL^−1^ of DNA). Insets within violins indicate mean (black dots) ± 1 SD; different letters above bars indicate significant differences among treatments (univariate ANOVA and post hoc Tukey test; *P* < 0.05, n = 10). Abbreviations: ANOVA, analysis of variance; CFU, colony-forming unit; FW, fresh weight; SD, standard deviation

To determine the effects of DNA treatment on the quality of the plant as a host for microbial pathogens, we aimed to take the perspective of the microbes, and therefore, we decided to quantify population densities reached at a specific time point after challenge as an approximation of microbial reproductive fitness [71–73]. Among four fungal strains tested, we detected no significant effect of DNA treatment on the density reached by *C. lindemuthianum* or *F. oxysporum* (Fig. 5a, GLM; *P* > 0.05, *n* = 10), whereas the necrotrophic fungi *B. cinerea* and *S. sclerotiorum* grew to significantly lower densities in DNA-treated plants as compared to control plants (Fig. 5a, GLM; *P* < 0.05, univariate analysis of variance [ANOVA], Tukey post hoc test; *P* < 0.05, *n* = 10). By contrast, all four bacterial strains tested (*Enterobacter* sp. strain FCB1*, P. syringae* pv*. phaseoli, P. syringae* pv*. syringae*, and *Xanthomonas phaseoli*) showed strongly reduced populations in DNA-treated plants as compared to controls (Fig. 5b, univariate ANOVA, Tukey post hoc test; *P* < 0.05, *n* = 10). Intriguingly though, post hoc tests revealed for all eight microbial strains that the population densities they reached when colonizing DNA-treated plants were similar among the three types of DNA; that means we found no significant differences between the self-DNA–treated and the nonself-DNA–treated plants (Fig. 5b). We conclude that the effects of exogenous DNA treatment on the quality of *P. vulgaris* as a host for microbial leaf pathogens are not self/nonself-specific—at least not if we compare DNA from different plant species.

Extracellular DNA can have direct antimicrobial activity, both in experimental setups and in natural settings (e.g., in plant DNA–containing extracellular nets that are secreted by plant roots to trap soil-borne pathogens) [67, 74]. To test for this possibility, we inoculated petri dishes with solid medium that had been pretreated with 100 µl of DNA at 50 µg mL^−1^ from each of the three plant species (*P. vulgaris*, *P. lunatus*, or *A. farnesiana*) and quantified bacterial CFUs 2 days after inoculation and fungal CFUs 4 days after inoculation. However, under these experimental conditions, we detected no significant effect of any type of plant DNA on any of the eight microbial strains (Fig. S1).

### The immune response to self-DNA enhances seed yield in the field

**Fig. 6:**
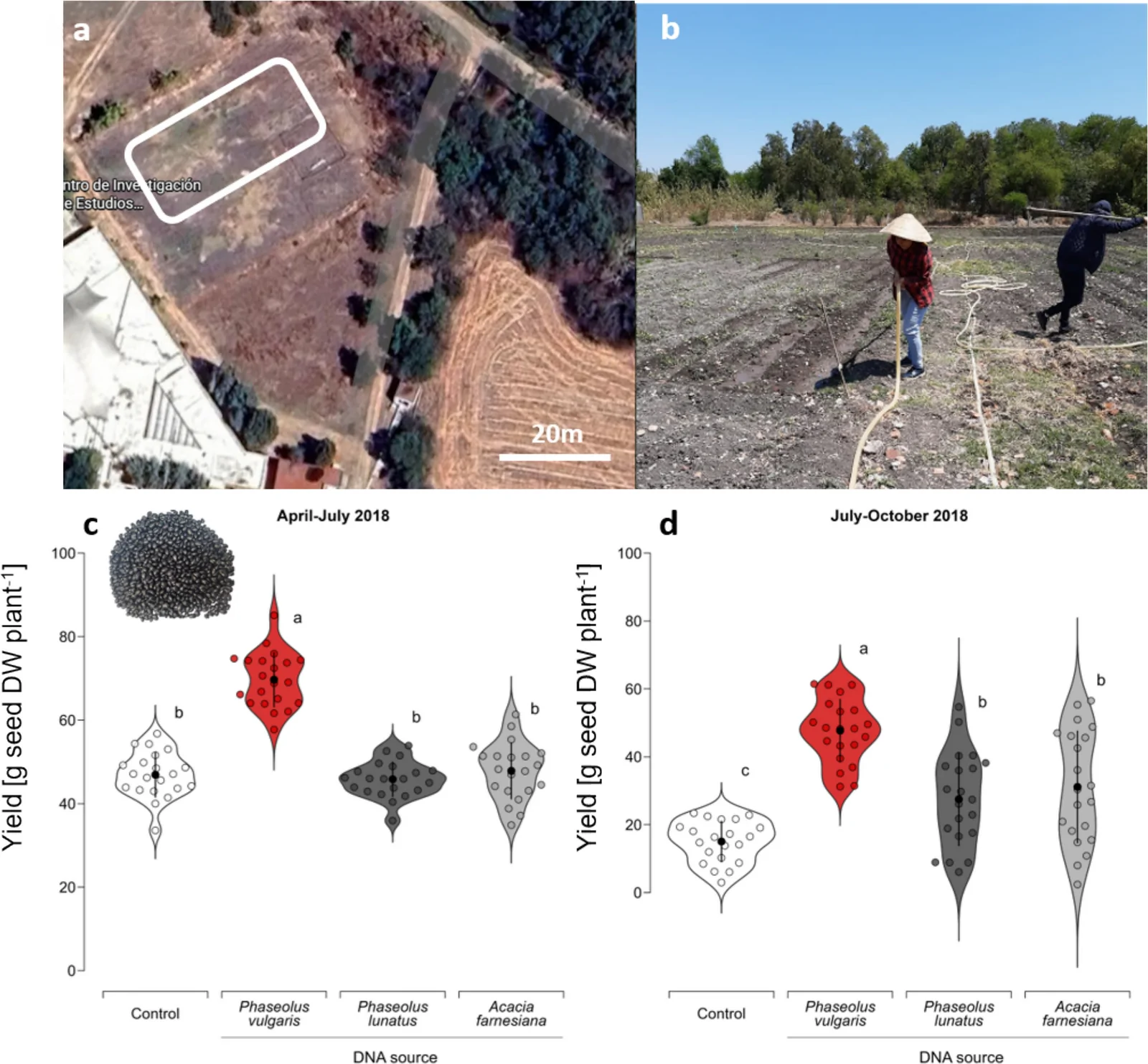
On-site images of fields and results of field experiment. Panel (**a**) is an aerial view of the field site (approx. 20° 43′ 13″ N and 101° 19′ 43″ W, 1730 m a.s.l.; screenshot taken from Google Maps* on July 12, 2026), and panel (**b**) shows the field being prepared for the experiment. Panels (**c**) and (**d**) are violin plots depicting the frequency distribution of the seed production (in grams of seed DW mass per individual) of field-grown *Phaseolus vulgaris* plants in (**c**) the rainy season and (**d**) the dry season that had been treated once (at an age of 4 weeks) with 50 µg mL^−1^ of self-DNA (*P. vulgaris* [red]) or nonself-DNA (*Phaseolus lunatus* [dark gray] and *Acacia farnesiana* [light gray]). The white violins represent the controls (0 µg mL^−1^ of DNA). Insets within violins indicate mean (black dots) ± SE; different letters above bars indicate significant differences among treatments (ANOVA and post hoc Fisher’s least significant difference test; *P* < 0.05, *n* = 3 blocks of seven individuals; see Fig. S2 for the arrangement of plants in each block). Abbreviations: ANOVA, analysis of variance; a.s.l., above sea level; DW, dry weight; SE, standard error. *https://www.google.com/maps/place/Centro+de+Investigaci%C3%B3n+y+de+Estudios+Avanzados+del+IPN,+Unidad+Irapuato+(Cinvestav)/@20.7206757,−101.3291263,182m/data=!3m1!1e3!4m6!3m5!1s0x842c8079b22da565:0xeb76ea6529839a98!8m2!3d20.7204173!4d-101.3291665!16s%2Fg%2F1wk6_2j_?entry=ttu&g_ep=EgoyMDI2MDQyMC4wIKXMDSoASAFQAw%3D%3D

The aforementioned reductions of herbivore-inflicted damage and pathogen populations indicate that exogenously applied self-DNA could be tool of a preventive pest control. However, ultimate effects of any plant resistance mechanism on yield are highly dependent on the environmental context [75, 76]. Therefore, we decided to quantify the effects of treatments with self- and nonself-DNA on seed yield of *P. vulgaris* plants grown under open field conditions, in both the rainy and the dry season (April-July and July–October, respectively). Bean seeds were sown directly into the soil, and the emerging plantlets were treated once with self- or nonself-DNA. Subsequently, the plants were allowed to finish their growth cycle without the application of any pesticide. We observed a significant treatment effect in both seasons (Fig. 6c and d, GLM; *P* < 0.05, *n* = 3 blocks of 7 individuals), although the magnitude and the self/nonself-specificity of the effects differed to some extent between the seasons. In the rainy season, treatment with self-DNA increased seed yield approximately 1.5-fold as compared to controls (Tukey post hoc test; *P* < 0.05), whereas treatment with nonself-DNA had no detectable effect (Fig. 6c). In the dry season, however, seed yield increased in response to all three DNA treatments (Tukey post hoc test; *P* < 0.05), although at different magnitudes (over threefold and approximately twofold in response to self- and nonself-DNA, respectively; see Fig. 6d).

## Discussion

Self-DNA triggers immune responses in plants and mammals [27, 77–79], yet with dramatically different outcomes: although humans usually develop severe inflammatory pathologies, critical illness, or autoimmune diseases, plants exhibit seemingly beneficial increases in their resistance to pests and diseases. Here, we asked whether this apparent lack of self-tolerance could be translated to the creation of a natural immunostimulant for crops. Indeed, self-DNA induced JA and SA production and increased the resistance to biotic stressors. A field experiment confirmed that a single treatment with self-DNA was sufficient to result in significantly higher yield in the rainy as well as the dry season. Strikingly, we observed a self-specific induction of JA, whereas SA was induced by both self and nonself-DNA. The response as shown by JA confirms the emerging general pattern: most studies that treated plants with DNA from different sources reported stronger immune responses to self-DNA than to nonself-DNA, and several studies showed a potential role of JA in this response [31–33, 50, 80–82]. Furthermore, a resistance induction by self-DNA to the necrotrophic fungus *B. cinerea* and the biotrophic bacterium *P. syringae* has already been reported for *A. thaliana* and tomato, and it could be related to JA signaling [32]. However, our results differ slightly from a recent report on a self/nonself-specific induction of SA in *A. thaliana* [83], leading us to conclude that the immune response to DNA is subject to interspecific differences that urgently require further investigation. If true, this situation would complicate any mechanistic understanding of the differential effects of self- versus nonself-DNA on plant innate immunity.

An accumulation of self-DNA in the extracellular space indicates massive cellular damage [84–86] and, therefore, it can be perceived as a damage-associated molecular pattern (DAMP), consistent with Matzinger’s danger model [18, 19]. Matzinger argues “that the immune system is more concerned with entities that do damage than with those that are foreign” [19], and suggests that damage is detected by monitoring the appearance of fragmented or mislocalized self-molecules. Self-DNA and other DAMPs similar to, for example, ATP and oligogalacturonides, usually trigger reactive oxygen species formation and induce JA, thereby inducing resistance to chewing herbivores and necrotrophic pathogens [85, 87–91]. In *P. vulgaris*, wounding and extracellular ATP increased reactive oxygen species formation and the activity of JA-inducible polyphenol oxidase [92]. The defense induction by plant-derived DNA also indicates that DNA is likely an active principle in the immunogenic effects of conspecific leaf homogenates [27, 69, 93, 94], which might also explain, at least in part, the success of multiple plant- or algae-derived biocontrol products [95].

The main motivation of the present work was to find out if the early immune responses to DNA benefit a plant that grows while under attack from pests or pathogens and whether this ultimately translates to increased yield. We observed phenotypic effects that were generally consistent with the responses of JA and SA. Self-DNA treatment induced JA and SA, reduced feeding by a chewing herbivore, and likewise the infection by four bacterial and two fungal pathogens: the biotrophic bacteria *P. syringae* pv*. phaseoli*, *P. syringae* pv*. syringae, X. axonopodis* pv. *phaseoli*, and necrotrophic bacterium *Enterobacter* sp. (a likely opportunistic pathogen that we had isolated from bean plants growing at the same site [60]); and the two necrotrophic fungi, *B. cinerea* and *S. sclerotiorum*. Nonself-DNA had no statistically significant effects on JA and did not trigger a significant resistance to the chewing herbivore, whereas its effects on SA and the colonization by all eight microbial pathogens were not distinguishable from those of self-DNA. Strikingly, we did not observe evidence for a trade-off between the JA- and the SA-signaling pathway since self-DNA induced both hormones to maximum levels.

Two pathogens were not detectably affected by any of the DNA treatments: *C. lindemuthianum* and *F. oxysporum*. Why the immune response to DNA did not translate to an increased resistance to these fungi remains unknown, although the hemibiotrophic lifestyle and the multiple strategies used by both fungi to suppress plant resistance provide possible explanations: hemibiotrophic pathogens switch from an initial biotrophic to a necrotrophic mode of infection and resistance to them hardly ever follows the classic JA/SA model. Correspondingly, various studies that examined the resistance to *F. oxysporum* of Arabidopsis mutants impaired in the accumulation of JA or SA reported enhanced susceptibility as well as enhanced resistance phenotypes [96], whereas other studies reported resistance to *Fusarium* in tomato that was completely independent of JA- or SA-signaling [97]. Similarly, transcriptomic responses in *P. vulgaris* during the compatible versus incompatible interaction with two different *C. lindemuthianum* strains were characterized mainly by differentially expressed genes that coincided between the two scenarios [98]. In summary, it seems safe to state that the induction of JA versus SA by self- and nonself-DNA treatments translated to similar patterns in the phenotypic resistance to an herbivore and eight microbial pathogens.

Moreover, these patterns are also likely to explain the observed yield improvements, at least in part. Unfortunately our experimental setup did not allow for excluding several alternative explanations, including (1) a fertilizing effect due to the supply of limited phosphorous, (2) growth promotion by DNA acting as a biostimulant as defined by the European Union [99], and (3) enhanced resilience to abiotic stress (e.g., drought stress). That plants can use DNA as a source of nutrients has been demonstrated by Paungfoo-Lonhienne and colleagues [100, 101], who showed that roots and pollen increased their growth in response to the uptake of exogenously applied DNA [100, 101]. However, we are not aware that this function also has been shown in leaves. Plant growth promotion by DNA seems, at the first glance, counterintuitive, particularly if we consider the inhibitory Mazzoleni effect and more recent studies showing that self-DNA triggers a cell cycle arrest, at least in Arabidopsis [37]. Nevertheless, the growth inhibition caused by self-DNA and other DAMPs is usually transient, and considering the successful use of the hormesis concept to interpret the effects of self-DNA in plants [102, 103], this option definitely merits further consideration. This is particularly true because showing a stimulating effect of self-DNA on plant nutrition and growth would strongly facilitate its registration as a biostimulant, at least in the European Union and in Mexico [99, 104].

Finally, the idea that self-DNA triggers enhanced resistance (or resilience) to abiotic stress gains some indirect support from the observation that the relative increase in yield was much more pronounced in the dry season than in the rainy season. In addition, unpublished observations by one author (M.H.) indicate that self-DNA treatment can increase the performance under drought not only in bean, but also in lettuce (*L. sativa*) and maize (*Z. mays*), and a transcriptomic study identified three genes belonging to the BAG family and AT1G66600 under the genes whose expression was specifically upregulated by self-DNA [37]; BAG genes control the induction of autophagy and have confirmed protective activities in response to drought and heat stress, and AT1G66600 is a WRKY transcription factor involved in drought tolerance [37].

In conclusion, our study supports a role of self-DNA as a DAMP in a plant’s immune response to wounding and infection, and it shows that the specific response to self-DNA can increase yield under field conditions, at least in common bean. We conclude that self-DNA merits attention as a tool for future, preventive strategies in the biological control of pests and plant diseases. However, much more work will be required to identify the molecular players that control the self/nonself-specific plant response to DNA and to explore its potential for the development of DNA-based biocontrol strategies.

## Supplementary Information

Supplementary Material 1. Fig. S1. No direct effects of plant DNA on fungal and bacterial pathogens. Violin plots depict the frequency distribution of CFUs of (a) the fungal pathogens *Botrytis cinerea*, *Colletotrichum lindemuthianum,* *Fusarium oxysporum*, and *Sclerotinia sclerotiorum*, and (b) the bacterial pathogens *Enterobacter* sp. strain FCB1, *P. syringae* pv. *phaseoli*, *P. syringae* pv.*syringae*, and *Xanthomonas axonopodis* pv. *phaseoli*, quantified 2 days for bacteria and 4 days for fungi, after inoculating petri dishes of solid medium that had been pretreated with 100 µl of 50 µg mL^−1^ fragmented plant DNA from *Phaseolus vulgaris* (red), *Phaseolus lunatus* (dark gray), or *Acacia farnesiana* (light gray). Insets within violins indicate mean (black dots) ± 1 SD; the absence of different letters indicates the absence of any significant differences among treatments (univariate ANOVA and post hoc Tukey test; *P* > 0.05, *n* = 6). Abbreviations: ANOVA, analysis of variance; CFU, colony-forming unit; FW, fresh weight; *P.*, *Pseudomonas*; SD, standard deviation.

Supplementary Material 2: Fig. S2. Spatial design of the field experiment. The spatial arrangement was of 12 blocks (3 per treatment) of 3 × 9 plants (27 plants each), in which plants of *P. vulgaris* were arranged in the experimental field plot at CINVESTAV Irapuato to avoid pseudoreplication. The distance was 35 cm between plants and 1 m between blocks. All 27 plants in each block received the same treatment, but only the inner seven plants (inside of the dotted lines) were used to quantify seed production. Abbreviations: *A.*, *Acacia*; *P.*, *Phaseolus*.

## Data Availability

The datasets generated during and/or analyzed during the current study are available from the corresponding author on reasonable request.
