# Supplementary material for: Self-DNA acts as an immunostimulant for common bean (Phaseolus vulgaris) that induces defense against pests and diseases and increases seed yield
Source: Crop Health. 2026 Sep 8;4(1):25. doi: 10.1007/s44297-026-00086-3 (PMC13554015; doi:10.1007/s44297-026-00086-3)
Supplement: Supplementary file 1 [file 44297_2026_86_MOESM2_ESM.docx]

**Fig. S2. Spatial design of the field experiment.** The spatial arrangement was of 12 blocks (3 per treatment) of 3 × 9 plants (27 plants each), in which plants of *P. vulgaris* were arranged in the experimental field plot at CINVESTAV Irapuato to avoid pseudoreplication. The distance was 35 cm between plants and 1 m between blocks. All 27 plants in each block received the same treatment, but only the inner seven plants (inside of the dotted lines) were used to quantify seed production. Abbreviations: *A.*, *Acacia*; *P.*, *Phaseolus*.
